# A New Belief Entropy in Dempster–Shafer Theory Based on Basic Probability Assignment and the Frame of Discernment

**DOI:** 10.3390/e22060691

**Published:** 2020-06-20

**Authors:** Jiapeng Li, Qian Pan

**Affiliations:** 1 School of Automation, Northwestern Polytechnical University, Xi’an 710072, China; 2 School of Electronics and Information, Northwestern Polytechnical University, Xi’an 710072, China

**Keywords:** Dempster–Shafer theory, basic probability assignment, frame of discernment, uncertainty measure, belief entropy, Shannon entropy

## Abstract

Dempster–Shafer theory has been widely used in many applications, especially in the measurement of information uncertainty. However, under the D-S theory, how to use the belief entropy to measure the uncertainty is still an open issue. In this paper, we list some significant properties. The main contribution of this paper is to propose a new entropy, for which some properties are discussed. Our new model has two components. The first is Nguyen entropy. The second component is the product of the cardinality of the frame of discernment (FOD) and Dubois entropy. In addition, under certain conditions, the new belief entropy can be transformed into Shannon entropy. Compared with the others, the new entropy considers the impact of FOD. Through some numerical examples and simulation, the proposed belief entropy is proven to be able to measure uncertainty accurately.

## 1. Introduction

How to measure uncertainty is a meaningful question to be solved. Our work will also discuss this issue. First of all, we need to know what uncertainty is.

The uncertainty problem is still very extensive. It means not certainly known, questionable, and problematic. Uncertainty can mainly divided into three types: vagueness, which is boundary uncertainty; nonspecificity, which is size (cardinality) uncertainty; and discord, which expresses conflict. Correspondingly, there are some theories to solve these problems: fuzzy set theory [1], probability theory [2], evidence theory [3,4], and rough sets [5]. Besides, some extended theories are also presented for the uncertainty measure, e.g., generalized evidence theory [6], complex numbers [7], fuzzy numbers [8,9,10], Z numbers [11,12], D numbers theory [13,14,15,16], and so on [17,18,19,20,21,22]. In this paper, we use evidence theory to study these open issues.

In 1960, Dempster [3] proposed upper and lower probabilities to solve the multivalued mapping problem. In 1971, Shafer [4] completed the theory proposed by Dempster and formed evidence theory, also called D-S theory. After years of exploration, D-S theory is a very effective tool for modeling and processing information uncertainty. In 1948, Shannon [23] used the concepts in thermodynamics to define information entropy. Under probability theory, Shannon entropy was very good at measuring the degree of information uncertainty. However, D-S theory is easier than probability theory for getting prior data, and the former has the advantage of fused data. Thus, we introduce D-S theory to replace probability theory in uncertainty.

D-S theory uses basic probability assignment (BPA), which is under the frame of discernment (FOD), to represent the degree of support for a focal element. Different FODs may have different BPAs. Besides, the core of D-S theory is Dempster’s combination rule. Dempster’s combination rule provides a way to fuse different BPAs. The proposition of evidence theory provides mathematical support for the establishment of uncertain models.

On the other hand, it is well known in information theory that Hartley [24] and Shannon’s measures are both effective ways to deal with information uncertainty. Meanwhile, D-S theory as an extension of probability theory contains much ignorance information. Thus, Höhle [25] was the earliest to combine D-S theory and Shannon entropy, namely Höhle entropy. Subsequently, Nguyen [26], Dubois and Prade [27], Klir [28], Jiroušek and Shenoy [29], Nikhil R. Pal [30,31], Deng [32], Pan and Deng [33], and Wang [34] defined their uncertainty models. Some of them have been successfully applied in real situations [35,36]. However, these models are not effective in some places [37,38].

According to their entropy, most of them are only focused on the BPA of every focal element and the cardinality of an element or use the belief function and plausibility function to measure uncertainty. Therefore, no one focused on FOD. Obviously, the scale of FOD can impact the degree of uncertainty. We combined different models and proposed a new uncertainty measurement, namely B& F entropy, because the uncertainty is determined by both BPA and FOD. In this method, it can well reflect the impact of FOD on uncertainty. In the end, we will give a few examples to compare the new model and others. Besides, we design a simulation to illustrate the feasibility and effectiveness of the proposed model.

The outline of the remainder of the paper is as follows. In Section 2, we briefly review the Hartley and Shannon measures and D-S theory. Some essential properties are briefly introduced in Section 3. Section 4 presents and existing uncertainty measures. In Section 5, we discuss some properties and define a new entropy. In Section 6, some significant numerical examples and simulations are carried out to illustrate the feasibility and effectiveness of the proposed belief entropy. In Section 7, we summarize our findings and conclude with some open questions.

## 2. Preliminaries

The focus of this paper is based on D-S theory and information entropy. We divide this section into two parts. In the D-S theory section, some basic concepts will briefly be introduced. In the information entropy section, we will introduce two typical representatives, the Hartley measure and Shannon entropy.

### 2.1. D-S Theory

Dempster–Shafer theory, also called evidence reasoning or evidence theory, originated from Dempster [3] and was developed by his student Shafer [4]. Through a series of improvements and reinforcements, a method of uncertainty reasoning using “evidence” and “combination” was formed. In a way, D-S theory is a promotion of Bayesian reasoning. Dempster–Shafer theory is often applied to pattern recognition [39,40,41,42,43,44], fault diagnosis [45,46,47,48], uncertainty modeling [20,49], clustering [50], decision making [51,52], risk analysis [53,54,55,56], and other hot fields [57,58].

The idea of D-S theory is based on the frame of discernment *X*, which $X=\{x_{1},x_{2}\cdots x_{n}\}$, and the set of all subsets in *X* is called the power set $2^{X}$. In the power set $2^{X}$, it contains $2^{\left|X\right|}$ elements. $\left|X\right|$ means the cardinality of *X*, which is the number of elements in *X*. Under this frame, Dempster and Shafer defined some basic concepts as follows.

#### 2.1.1. Basic Belief Assignment

Based on the above power set $2^{X}$, function *m* :$2^{X}\to \left[0,1\right]$ satisfies:

(1) $$\begin{array}{c} \left\{\begin{array}{c} \sum_{a\in 2^{X}}m\left(a\right)=1\\ m(\emptyset )=0 \end{array}\right. \end{array}$$

The function m(a) is also called a basic probability assignment or mass function. If m(a)>0, then *a* is a focal element. m(a) means the value of trust that the object belongs to *a*. The larger m(a) is, the higher the trust value is.

Some definitions of BPA are as follows. The vacuous BPA means entirely unknown for the true result. In contrast, from the Bayesian BPA, we can know to which category the target should belong.

#### 2.1.2. Belief Function

The belief function is the sum of the basic probability assignments for all subsets of *a* and is given by:

(2) $$Bel\left(a\right)=\sum_{b\subseteq a}m\left(b\right),\forall a\in 2^{X}$$

It is the lower limit of support for *a*.

#### 2.1.3. Plausibility Function

The plausibility function is the sum of the basic probability assignments for all subsets that intersect with *a* and is given by:

(3) $$Pl\left(a\right)=\sum_{b\cap a=\emptyset }m\left(b\right),\forall a\in 2^{X}$$

It is the upper limit of support for *a*.

The value, between the belief function and plausibility function, is the degree of uncertainty for evidence.

#### 2.1.4. Dempster’s Combination Rule

Dempster’s combination rule is the most commonly used method in evidence fusion. This rule takes into account the degree of conflict between the evidence and defined conflict coefficient *k* to measure the degree of conflict among different evidence.

Suppose $m_{1}$ and $m_{2}$ are independent BPAs from the different evidence resources, respectively. The fusion result of $m_{1}$ and $m_{2}$ under Dempster’s combination is as follows:

(4) $$\left\{\begin{array}{c} m(a)=0,a=\emptyset \\ m\left(a\right)=\frac{\sum_{b\cap c=a}m_{1}\left(b\right)m_{2}\left(c\right)}{1-k},a\neq \emptyset \end{array}\right.$$

where *k* is a conflict coefficient, defined by:

(5) $$k=\sum_{b\cap c=\emptyset }m_{1}\left(b\right)m_{2}\left(c\right)$$

Notice that Dempster’s combination rule is invalid, if two bodies of evidence completely conflict (k=1). Furthermore, if k>1, Dempster’s combination rule cannot be applied to the two BPAs’ fusion.

### 2.2. Origin of Information Entropy

Different authors have measured information uncertainty in a variety of ways, and Hartley and Shannon laid the foundation for it. The information entropy and their extended models have been applied to many fields [59]. Next, we will briefly introduce the Hartley measure and Shannon entropy.

#### 2.2.1. Hartley Measure

Suppose *X* is an FOD and *a* is a subset of *X*. Then, the Hartley measure [24] is defined as:

(6) $$H\left(a\right)=log_{2}\left|a\right|$$

where $\left|a\right|$ means the cardinality of *a*.

Obviously, the measurement is proportional to the cardinality of *a*. When *a* is a singleton of *X*, H(a)=0, this means there is no conflict. Unfortunately, the measurement method of Hartley does not show the effect of the probability distribution on the degree of uncertainty.

#### 2.2.2. Shannon Entropy

In 1948, Shannon [23] proposed information entropy, namely Shannon entropy. His model uses the concept of entropy from thermodynamics.

(7) $$H\left(x\right)=\sum_{x\in X}P\left(x\right)log_{2}\frac{1}{P(x)}$$

where P(x) is the probability of *x* and P(x) satisfies $\sum_{x\in X}P\left(x\right)=1$.

As he said in his thesis, the role of information is used to eliminate the uncertainty. Shannon entropy is an excellent way to measure and eliminate uncertainty. It played a crucial role in solving the probability problem. We can conclude from his definition that it is based on the probability distribution. With the emergence of D-S theory, the information entropy was given a new meaning. The format of our new model is also derived from the Shannon entropy.

## 3. Properties of the Uncertainty Measure in D-S Theory

According to Klir and Wierman [60] and Klir and Folger [61], we introduce some important properties of entropy for D-S theory, including non-negativity, maximum, monotonicity, probability consistency, additivity, sub-additivity, and range. These properties for a measure that captures both discord and non-specificity are defined as follows.

### 3.1. Non-Negativity

Suppose *m* is a BPA on FOD *X*; the entropy H(m) must be:

(8) H(m)>0

where this is equality if and only if m(x)=1 and x∈X.

Only when entropy satisfies the non-negativity property, it provides a standard for measurement uncertainty.

### 3.2. Maximum Entropy

It makes sense that the vacuous BPA $m_{v}$ for uncertainty is lager than other normal BPAs $m_{n}$. Thus, the maximum entropy property is defined as:

(9) $$H\left(m_{v}\right)>H\left(m_{n}\right)$$

### 3.3. Monotonicity

As the number of focal elements in FOD increases, so should the degree of uncertainty. The monotonicity property is defined as:

(10) $$H\left(m_{X}\right)>H\left(m_{Y}\right)$$

where $m_{X}$ and $m_{Y}$ are the vacuous BPAs for FOD *X* and FOD *Y*. Meanwhile, $\left|X\right|>\left|Y\right|$.

### 3.4. Probability Consistency

Let $m_{B}$ be a Bayesian BPA, and then, the entropy should be the same as Shannon entropy. Therefore, the probability consistency property follows as:

(11) $$H\left(m_{B}\right)=H_{S}\left(P_{X}\right)=\sum_{x\in X}P_{X}\left(x\right)log_{2}\frac{1}{P_{X}\left(x\right)}$$

where $H_{S}$ is the Shannon entropy and $P_{X}$ is the BPA of *X* corresponding to $m_{b}$.

### 3.5. Additivity

Let $m_{X}$ and $m_{Y}$ be independent BPAs on FOD *X* and FOD *Y*, respectively. ⊕ means Dempster’s combination rule. Thus, the additivity property is defined as:

(12) $$H\left(m_{X}⊕m_{Y}\right)=H\left(m_{X}\right)+H\left(m_{Y}\right)$$

where $m_{X}⊕m_{Y}$ is a BPA for FOD {X,Y}. Note that $m\left(a\times b\right)=m_{X}\left(a\right)m_{Y}\left(b\right)$, where *m* is the $m_{X}$ and $m_{Y}$ combined by Dempster’s combination rule.

### 3.6. Sub-Additivity

Let *m* be a BPA on FOD {X×Y}. Let $m^{↓X}$ and $m^{↓Y}$ be the marginal BPAs of FOD *X* and FOD *Y*. Then, define:

(13) $$H\left(m\right)\leq H\left(m^{↓X}\right)+H\left(m^{↓Y}\right)$$

### 3.7. Range

As Klir and Wierman defined, the range of H(m) is $\left[0,log_{2}\left|X\right|\right]$.

## 4. The Development of Entropy Based on D-S Theory

In this section, some belief entropies of BPAs in D-S theory proposed by others are reviewed. We also discuss whether or not these models satisfy the properties we list.

Yager [62] defined the belief entropy using the conflict coefficient between two focal elements, simplified as follows:

(14) $$H_{Y}\left(m\right)=-\sum_{x\in 2^{X}}m\left(a\right)log_{2}Pl\left(a\right)$$

where Pl(a) is the plausibility function associated with *a* under *m*. The entropy of Yager only measures the degree of conflict between evidence. $H_{Y}\left(m\right)$ only satisfies the additivity property.

Dubois [27] used a new information measurement method to get the new formula of entropy.

(15) $$H_{D}\left(m\right)=\sum_{a\in 2^{X}}m\left(a\right)log_{2}\left|a\right|$$

From the definition of Dubois, this entropy only answers the question of the non-specific part of the uncertainty. If *m* is a Bayesian BPA, then $H_{D}\left(m\right)=0$. It is noticeable that $H_{D}\left(m\right)$ is clearly a weighted Hartley [24] measure. $H_{D}\left(m\right)$ satisfies the maximum entropy and monotonicity properties.

Nguyen [26] defined a new entropy according to Shannon entropy.

(16) $$H_{N}\left(m\right)=\sum_{a\in 2^{X}}m\left(a\right)log_{2}\frac{1}{m(a)}$$

From the definition format, it only uses the BPA to capture the part of the conflict. This is inaccurate for uncertain measurements. It only satisfies the probabilistic consistency property and the additivity property.

Lamata and Moral [63] used the entropy theory proposed by Yager and Dubois.

(17) $$\begin{array}{cc} H_{L\&M}\left(m\right) & =H_{Y}\left(m\right)+H_{D}\left(m\right)\\ \; & =\sum_{a\in 2^{X}}m\left(a\right)log_{2}\frac{\left|a\right|}{Pl(a)} \end{array}$$

They both have two components: one measures the innate contradiction, while the other measures the imprecision of the information. This definition does not satisfy the maximum entropy and sub-additivity properties.

Jiroušek and Shenoy [29] entropy is a combination of the Shannon and Dubois definitions.

(18) $$\begin{array}{cc} H_{J\&S}\left(m\right) & =H_{S}\left(Pl\_P_{m}\right)+H_{D}\left(m\right)\\ \; & =\sum_{x\in X}Pl\_P_{m}log_{2}\frac{1}{Pl\_P_{m}}+\sum_{a\in 2^{X}}m\left(a\right)log_{2}\left|a\right| \end{array}$$

where $Pl\_P_{m}$ is the normalized result of plausibility function $Pl_{m}$. The first part is the measurement of conflict based on Shannon entropy, and the second part is to measure the non-specificity portion of uncertainty. The entropy of $H_{J\&S}\left(m\right)$ satisfies non-negativity, maximum entropy, monotonicity, probability consistency, and additivity.

Klir and Ramer [28] defined:

(19) $$H_{K\&R}\left(m\right)=-\sum_{a\in 2^{X}}m\left(a\right)log_{2}\sum_{b\in 2^{X}}m\left(b\right)\frac{\left|b-a\right|}{\left|b\right|}$$

Due to the Yager entropy not concluded the broader view of conflict (it only considered the conflict situation of B∩A=∅), Klir and Ramer proposed a new method to solve this problem. It is easy to see that this entropy can measure the conflict of evidential claims within each body of evidence in bits. However, under certain conditions, it is difficult for $H_{K\&R}\left(m\right)$ to express the aspect of uncertainty. It just does not satisfy the maximum entropy property.

Nikhil R. Pal [30,31] focused on nonspecificity and randomness under a total uncertainty environment.

(20) $$\begin{array}{cc} H_{P}\left(m\right) & =\sum_{a\in 2^{X}}m\left(a\right)log_{2}\frac{\left|a\right|}{m(a)}\\ \; & =\sum_{a\in 2^{X}}m\left(a\right)log_{2}\frac{1}{m(a)}+\sum_{a\in 2^{X}}m\left(a\right)log_{2}\left|a\right| \end{array}$$

They summed up the methods proposed by Lamata and Moral and Klir and Ramer. It was pointed out that there would be mistakes against common sense in certain situations. The first part is, in some sense, analogous to Yager’s entropy, and the second part measures the conflict of the body of evidence. It does not satisfy the maximum entropy property.

Jousselme [64] entropy is based on pignistic transformation $BetP_{m}\left(a\right)$ [65].

(21) $$H_{J}\left(m\right)=\sum_{a\in 2^{X}}BetP_{m}\left(a\right)log_{2}\frac{1}{BetP_{m}\left(a\right)}$$

He finally proved that as the evidence changes, the entropy becomes more sensitive.

Deng [32] defined an entropy:

(22) $$H_{Deng}\left(m\right)=\sum_{a\in 2^{X}}m\left(a\right)log_{2}\left[\frac{2^{\left|a\right|}-1}{m(a)}\right]$$

As proven by Joaquín Abellán [66], the Deng entropy does not satisfy the monotonicity, additivity, and subadditivity properties.

Pan and Deng [33] developed Deng entropy and defined it as follows:

(23) $$H_{Bel}\left(m\right)=-\sum_{a\in 2^{X}}\frac{Bel(a)+Pl(a)}{2}log_{2}\frac{Bel(a)+Pl(a)}{2(2^{\left|a\right|}-1)}$$

where Bel(a) and Pl(a) are the belief function and plausibility function, respectively. $H_{Bel}\left(m\right)$ uses the interval probability to measure the discord and non-specificity uncertainty of BPA. It does not satisfy the maximum entropy, additivity, sub-additivity, and range properties.

W [34] is another modified model based on Deng entropy:

(24) $$H_{W}\left(m\right)=-\sum_{a\in X}m\left(a\right)log_{2}\left(\frac{m(a)}{2^{\left|a\right|}-1}{\left(1+\epsilon \right)}^{f(\left|X\right|)}\right)$$

where ϵ is a constant and ϵ≥0, $f(\left|X\right|)$ is the function about the cardinality of *X*. ϵ is a change number; it can take different values to represent different entropies. However, as the parameter ϵ changes, it has little effect on the value of W entropy [34].

## 5. A New Belief Entropy Based on Evidence Theory

As introduced at the start of the first chapter of Shafer’s book [4], D-S theory is a theory of evidence. That means using the mathematical form to express the degree of support for evidence.

Based on the entropy proposed by previous scholars, for the measurement method of information uncertainty, there remain several aspects of the frame of discernment about which relatively little is known. In D-S theory, if we have the same cardinality of BPA, but different FODs, the results of uncertainty should be changed. However, most of them we listed above only focused on the value of BPA or the cardinality of every BPA, and the effect of FOD was totally ignored. Thus, these definitions cannot measure the degree of uncertainty under different FOD. To improve these deficiencies, we suggest that the FOD is also important for the measurement of uncertainty. Therefore, we introduce the scale of FOD to our new entropy. The new belief entropy based on D-S theory, namely B& F entropy, is defined as follows:

(25) $$H_{B\&F}\left(m\right)=\sum_{a\in 2^{X}}m\left(a\right)log_{2}\frac{{\left|a\right|}^{\left|X\right|}}{m(a)}$$

where $\left|a\right|$ denotes the cardinality of the focal element *a* and $\left|X\right|$ equals the number of elements in FOD.

Like some of the definitions we mentioned, the new definition can be represented by a combination of other entropies. Thus, the new entropy also can be expressed as:

(26) $$\begin{array}{cc} H_{B\&F}\left(m\right) & =\sum_{a\in 2^{X}}m\left(a\right)log_{2}\frac{1}{m(a)}+\sum_{a\in 2^{X}}m\left(a\right)log_{2}{\left|a\right|}^{\left|X\right|}\\ \; & =H_{N}\left(m\right)+\left|X\right|H_{D}\left(m\right) \end{array}$$

where $H_{N}\left(m\right)$ is Nguyen’s entropy and $H_{D}\left(m\right)$ is Dubois’ entropy. Obviously, the new entropy is a combination of $H_{N}\left(m\right)$ and $\left|X\right|$ times $H_{D}\left(m\right)$. Similar to most of the belief entropies, the first component $\sum_{a\in 2^{X}}m\left(a\right)log_{2}m^{-1}\left(a\right)$ in the new belief entropy is designed to measure the discord uncertainty of BPA. At length, the second component $\sum_{a\in 2^{X}}m\left(a\right)log_{2}{\left|a\right|}^{\left|X\right|}$ is the measure of non-specificity of the mass function among various focal elements [27,32,61]. In addition, it can capture the information about the size of cardinality. When *m* is a Bayesian BPA or the cardinality of FOD equals one, the new entropy degenerates to Pal’s definition.

The most important information about FOD is the quantity of the focal element, namely $\left|X\right|$. If $\left|X\right|$ is modified, the accuracy of uncertain measurement will be affected. Here, we use an example to show that $\left|X\right|$ is the best way to represent the information of FOD.

**Figure 1:**
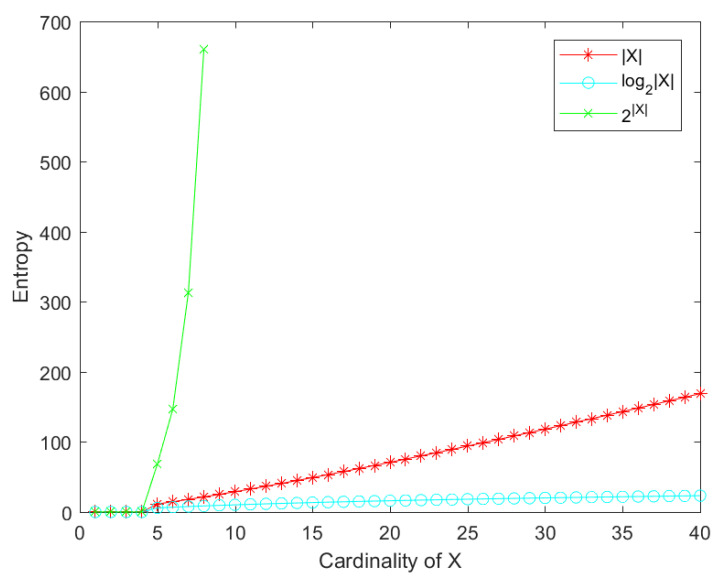
Comparison of different frame of discernment (FOD) information.

As shown in Figure 1, it is obvious that $log_{2}\left|X\right|$ and $2^{\left|X\right|}$ cannot reflect the effect of FOD on entropy very well. When the cardinality of FOD is greater than 10, $log_{2}\left|X\right|$ is almost constant, but $2^{\left|X\right|}$ is very large. Thus, $\left|X\right|$ can well contain the information of the FOD size.

The new entropy connects the degree of information uncertainty and the FOD, meanwhile improving the information uncertainty measurement method.

According to Section 3, the basic properties of the new belief entropy are proven as follows:

(P1) Non-negativity:

Let $\left|a\right|$ be a cardinality of the focal element and $\left|X\right|$ be a cardinality of FOD. It is obvious that ${\left|a\right|}^{\left|X\right|}>1$; thus, $H_{B\&F}\left(m\right)\geq 0$, if and only if *m* is the Bayesian BPA and m=1. Therefore, the new definition satisfies the non-negativity property.

(P2) Maximum entropy:

Let $m_{b}$ be a Bayesian BPA and $m_{v}$ be a vacuous BPA, then $H_{B\&F}\left(m_{b}\right)=log_{2}\left|X\right|$, $H_{B\&F}\left(m_{v}\right)=\left|X\right|log_{2}\left|X\right|$. Although, according to our calculations, $H_{B\&F}\left(m_{v}\right)>H_{B\&F}\left(m_{b}\right)$, it does not mean $H_{B\&F}\left(m_{v}\right)$ is the maximum value. Later, we will further explain the max value through simulation. In this part, we just give some simple explanations.

From the definition of Nguyen we introduced, this entropy does not satisfy the maximum entropy, as it consists of Nguyen’s entropy and Dubois entropy. Thus, the maximum entropy is not satisfied with the new belief entropy.

(P3) Monotonicity:

We suppose that $m_{v}$ denotes the vacuous BPA, then $H_{B\&F}\left(m_{v}\right)=\left|X\right|log_{2}\left|X\right|$. Obviously, $H_{B\&F}\left(m_{v}\right)$ increases with $\left|X\right|$. Therefore, $H_{B\&F}\left(m\right)$ satisfies the monotonicity property.

(P4) Probability consistency:

When $m_{b}$ is a Bayesian BPA, then $\left|a\right|=1$, $H_{B\&F}\left(m\right)=\sum_{a\in 2^{X}}m\left(a\right)log_{2}\frac{1}{m(a)}$. From this result, we conclude that the new belief entropy satisfies the probability consistency property.

(P5) Additivity and sub-additivity:

Let $c=a\times b\in 2^{X\times Y}$, where *a*, *b*, *c* is a focal element and *X*, *Y* means the FOD. Meanwhile, $a\in 2^{X}$ and $b\in 2^{Y}$. According to the definition of the above properties, $m\left(c\right)=m^{↓X}\left(a\right)\times m^{↓Y}\left(b\right)$, where $m^{↓X}$ is the marginal BPA for *X* and $m^{↓Y}$ is the marginal BPA for *Y*.

$$\begin{array}{cc} \; & H_{B\&F}\left(c\right)=\sum_{c\in 2^{X\times Y}}m\left(c\right)log_{2}\frac{{\left|c\right|}^{\left|X\right|\left|Y\right|}}{m(c)}\\ \; & =\sum_{\begin{array}{c} a\in 2^{X}\\ b\in 2^{Y} \end{array}}m^{↓X}\left(a\right)m^{↓Y}\left(b\right)log_{2}{\left(\left|a\right|\left|b\right|\right)}^{\left|X\right|\left|Y\right|}-\sum_{\begin{array}{c} a\in 2^{X}\\ b\in 2^{Y} \end{array}}m^{↓X}\left(a\right)m^{↓Y}\left(b\right)log_{2}\left(m^{↓X}\left(a\right)m^{↓Y}\left(b\right)\right)\\ \; & =\sum_{\begin{array}{c} a\in 2^{X}\\ b\in 2^{Y} \end{array}}m^{↓X}\left(a\right)m^{↓Y}\left(b\right)log_{2}{\left|a\right|}^{\left|X\right|\left|Y\right|}+\sum_{\begin{array}{c} a\in 2^{X}\\ b\in 2^{Y} \end{array}}m^{↓X}\left(a\right)m^{↓Y}\left(b\right)log_{2}{\left|b\right|}^{\left|X\right|\left|Y\right|}\\ \; & -\sum_{\begin{array}{c} a\in 2^{X}\\ b\in 2^{Y} \end{array}}m^{↓X}\left(a\right)m^{↓Y}\left(b\right)log_{2}m^{↓X}\left(a\right)-\sum_{\begin{array}{c} a\in 2^{X}\\ b\in 2^{Y} \end{array}}m^{↓X}\left(a\right)m^{↓Y}\left(b\right)log_{2}m^{↓X}\left(b\right)\\ \; & =\sum_{b\in 2^{Y}}m^{↓Y}\left(b\right)\sum_{a\in 2^{X}}m^{↓X}\left(a\right)log_{2}{\left|a\right|}^{\left|X\right|\left|Y\right|}+\sum_{a\in 2^{X}}m^{↓X}\left(a\right)\sum_{b\in 2^{Y}}m^{↓Y}\left(b\right)log_{2}{\left|b\right|}^{\left|X\right|\left|Y\right|}\\ \; & -\sum_{b\in 2^{Y}}m^{↓Y}\left(b\right)\sum_{a\in 2^{X}}m^{↓X}\left(a\right)log_{2}m^{↓X}\left(a\right)-\sum_{a\in 2^{X}}m^{↓X}\left(a\right)\sum_{b\in 2^{Y}}m^{↓Y}\left(b\right)log_{2}m^{↓Y}\left(b\right)\\ \; & =\sum_{a\in 2^{X}}m^{↓X}\left(a\right)log_{2}{\left|a\right|}^{\left|X\right|\left|Y\right|}+\sum_{b\in 2^{Y}}m^{↓Y}\left(b\right)log_{2}{\left|b\right|}^{\left|X\right|\left|Y\right|}\\ \; & -\sum_{a\in 2^{X}}m^{↓X}\left(a\right)log_{2}m^{↓X}\left(a\right)-\sum_{b\in 2^{Y}}m^{↓Y}\left(b\right)log_{2}m^{↓Y}\left(b\right)\\ \; & =\sum_{a\in 2^{X}}m^{↓X}\left(a\right)log_{2}\frac{{\left|a\right|}^{\left|X\right|\left|Y\right|}}{m^{↓X}\left(a\right)}+\sum_{b\in 2^{Y}}m^{↓Y}\left(b\right)log_{2}\frac{{\left|b\right|}^{\left|X\right|\left|Y\right|}}{m^{↓Y}\left(b\right)}\\ \; & \geq \sum_{a\in 2^{X}}m^{↓X}\left(a\right)log_{2}\frac{{\left|a\right|}^{\left|X\right|}}{m^{↓X}\left(a\right)}+\sum_{b\in 2^{Y}}m^{↓Y}\left(b\right)log_{2}\frac{{\left|b\right|}^{\left|Y\right|}}{m^{↓Y}\left(b\right)}\\ \; & =H_{B\&F}\left(m^{↓X}\left(a\right)\right)+H_{B\&F}\left(m^{↓Y}\left(b\right)\right) \end{array}$$

We can see from the above proof that the new entropy satisfies the additivity property, if and only if $\left|X\right|=\left|Y\right|=1$. Otherwise, the new belief entropy neither satisfies the additivity property nor sub-additivity.

To be more intuitive, we consider the following example:

Let *Z* be the product of FOD $X=\{x_{1},x_{2}\}$ and FOD $Y=\{y_{1},y_{2},y_{3}\}$. We have that BPA on *Z* is *m*, and the marginal BPAs on *X* and *Y* are $m^{↓X}$ and $m^{↓Y}$. We suppose the case on *Z* is shown as follows:

$$m\left(\left\{z_{11}\right\}\right)=0.1,m\left(\left\{z_{13}\right\}\right)=0.1$$

$$m\left(\left\{z_{12}\right\}\right)=0.1,m(\left\{\left(z_{21}\right\}\right)=0.1$$

$$m\left\{\left(z_{22}\right\}\right)=0.2,m\left(\left\{z_{23}\right\}\right)=0.2$$

$$m\left(Z\right)=1-m\left(\left\{z_{11}\right\}\right)-m\left(\left\{z_{13}\right\}\right)-m\left(\left\{z_{12}\right\}\right)-m\left(\left\{z_{21}\right\}\right)-m\left(\left\{z_{22}\right\}\right)-m\left(\left\{z_{23}\right\}\right)=0.2$$

where $Z_{i,j}=\left\{x_{i},y_{j}\right\}$. Thus, the BPAs on *X* and *Y* are:

$$m^{↓X}\left(\left\{x_{1}\right\}\right)=0.3,m^{↓X}\left(\left\{x_{2}\right\}\right)=0.5$$

$$m^{↓X}\left(X\right)=1-m^{↓X}\left(\left\{x_{1}\right\}\right)-m^{↓X}\left(\left\{x_{2}\right\}\right)=0.2$$

$$m^{↓Y}\left(\left\{y_{1}\right\}\right)=0.2,m^{↓Y}\left(\left\{y_{2}\right\}\right)=0.3,m^{↓Y}\left(\left\{y_{3}\right\}\right)=0.3$$

$$m^{↓Y}\left(Y\right)=1-m^{↓Y}\left(\left\{y_{1}\right\}\right)-m^{↓Y}\left(\left\{y_{2}\right\}\right)-m^{↓Y}\left(\left\{y_{3}\right\}\right)=0.2$$

The calculation results are as follows:

$$\begin{array}{cc} \; & \left|Z\right|=6,\left|X\right|=2,\left|Y\right|=3\\ \; & H_{B\&F}\left(m\right)=\sum_{z\in 2^{X\times Y}}m\left(z\right)log_{2}\frac{{\left|z\right|}^{\left|X\right|\left|Y\right|}}{m(z)}\\ \; & =2\times 0.1\times log_{2}\frac{1^{6}}{0.1}+2\times 0.1\times log_{2}\frac{1^{6}}{0.1}+2\times 0.2\times log_{2}\frac{1^{6}}{0.2}+0.2\times log_{2}\frac{6^{6}}{0.2}\\ \; & =0.6644+0.6644+0.9288+3.5663\\ \; & =5.8239\\ \; & H_{B\&F}\left(m^{↓X}\right)=\sum_{a\in 2^{X}}m^{↓X}\left(a\right)log_{2}\frac{{\left|a\right|}^{\left|X\right|}}{m^{↓X}\left(a\right)}\\ \; & =0.3\times log_{2}\frac{1^{2}}{0.3}+0.5\times log_{2}\frac{1^{2}}{0.5}+0.2\times log_{2}\frac{2^{2}}{0.2}\\ \; & =0.5211+0.5+0.8644\\ \; & =1.8855\\ \; & H_{B\&F}\left(m^{↓Y}\right)=\sum_{b\in 2^{Y}}m^{↓Y}\left(b\right)log_{2}\frac{{\left|b\right|}^{\left|Y\right|}}{m^{↓Y}\left(b\right)}\\ \; & =0.2\times log_{2}\frac{1^{3}}{0.2}+0.3\times log_{2}\frac{1^{3}}{0.3}+0.3\times log_{2}\frac{1^{3}}{0.3}\\ \; & +0.2\times log_{2}\frac{3^{3}}{0.2}\\ \; & =0.4644+0.5211+0.5211+1.4154\\ \; & =2.9220\\ \; & H_{B\&F}\left(m^{↓X}\right)+H_{B\&F}\left(m^{↓Y}\right)=1.8855+2.9220=4.8075 \end{array}$$

Obviously, $H_{B\&F}\left(m\right)>H_{B\&F}\left(m^{↓X}\right)+H_{B\&F}\left(m^{↓Y}\right)$. Therefore, the additivity and sub-additivity properties are not satisfied with the new entropy.

(P6) Range:

As demonstrated by the maximum entropy property, the value of the new entropy $H_{B\&F}\left(m_{v}\right)=\left|X\right|log_{2}\left|X\right|$, and $H_{B\&F}\left(m_{v}\right)>log_{2}\left|X\right|$. Thus, it does not satisfy the range property.

From the above results we proved, the new belief entropy satisfies the non-negativity, monotonicity, and probability consistency properties, and does not satisfy the maximum entropy, additivity, subadditivity, and range properties.

## 6. Numerical Example and Simulation

In the first part of this section, some examples are given to illustrate the effectiveness of the new belief entropy. The influence of different BPAs on B&F entropy is shown in the second section.

### 6.1. Numerical Example

#### 6.1.1. Example 1

Let FOD X={a}, and we get a BPA from the sensor as m({a})=1. Shannon entropy and the new definition proposed by the authors’ calculation results are as follows:

$$H_{B\&F}\left(m\right)=H_{S}\left(m\right)=1\times log_{2}1=0$$

#### 6.1.2. Example 2

Suppose there are three FODs $X_{1}=\left\{x_{1},x_{2}\right\}$, $X_{2}=\left\{x_{1},x_{2},x_{3},x_{4}\right\}$, $X_{3}=\left\{x_{1},x_{2},x_{3},x_{4},x_{5}\right\}$. Every Bayesian BPA $m_{1},m_{2},m_{3}$ of these FODs is equal. Their BPAs are as follows:

$$m_{1}\left(\left\{x_{1}\right\}\right)=m_{1}\left(\left\{x_{2}\right\}\right)=0.5$$

$$m_{2}\left(\left\{x_{1}\right\}\right)=m_{2}\left(\left\{x_{2}\right\}\right)=m_{2}\left(\left\{x_{3}\right\}\right)=m_{2}\left(\left\{x_{4}\right\}\right)=0.25$$

$$m_{3}\left(\left\{x_{1}\right\}\right)=m_{3}\left(\left\{x_{2}\right\}\right)=m_{3}\left(\left\{x_{3}\right\}\right)=m_{3}\left(\left\{x_{4}\right\}\right)=m_{3}\left(\left\{x_{5}\right\}\right)=0.2$$

The new belief entropy is calculated as follows:

$$H_{B\&F}\left(m_{1}\right)=2\times 0.5\times log_{2}\frac{1^{2}}{0.5}=1$$

$$H_{B\&F}\left(m_{2}\right)=4\times 0.25\times log_{2}\frac{1^{4}}{0.25}=2$$

$$H_{B\&F}\left(m_{3}\right)=5\times 0.2\times log_{2}\frac{1^{5}}{0.2}=2.3219$$

It is obvious that uncertainty increases as the number of focal elements increases. This is reasonable.

#### 6.1.3. Example 3

Using the FOD raised by Example 2 and the vacuous BPAs $m_{1}\left(\left\{x_{1},x_{2}\right\}\right)=1$, $m_{2}\left(\left\{x_{1},x_{2},x_{3},x_{4}\right\}\right)=1$, $m_{3}\left(\left\{x_{1},x_{2},x_{3},x_{4},x_{5}\right\}\right)=1$, the new entropy results are calculated as follows:

$$H_{B\&F}\left(m_{1}\right)=1\times log_{2}\frac{2^{2}}{1}=2$$

$$H_{B\&F}\left(m_{2}\right)=1\times log_{2}\frac{4^{4}}{1}=8$$

$$H_{B\&F}\left(m_{3}\right)=1\times log_{2}\frac{5^{5}}{1}=11.6096$$

Comparing Example 2 and Example 3, it is easy to get that the results of the vacuous BPA are bigger than the results of the Bayesian BPA.

#### 6.1.4. Example 4

In this example, we compare the difference between Pal entropy and B&F entropy. Let FOD $X_{1}=\left\{x_{1},x_{2},x_{3},x_{4},x_{5}\right\}$ and $X_{2}=\left\{x_{1},x_{2},x_{3},x_{4}\right\}$. Meanwhile, suppose the following two situations exist:

$$C_{1}:m_{1}\left(\left\{x_{1},x_{2},x_{3}\right\}\right)=0.3,m_{1}\left(\left\{x_{4},x_{5}\right\}\right)=0.7$$

$$C_{2}:m_{2}\left(\left\{x_{1},x_{2},x_{3}\right\}\right)=0.3,m_{2}\left(\left\{x_{3},x_{4}\right\}\right)=0.7$$

Thus, the Pal entropy and B&F entropy results calculated and compared are the following:

$$\begin{array}{cc} C_{1}:H_{B\&F}\left(m_{1}\right) & =0.3\times log_{2}\frac{3^{5}}{0.3}+0.7\times log_{2}\frac{2^{5}}{0.7}\\ \; & =2.8985+3.8602=6.7587\\ C_{1}:H_{P}\left(m_{1}\right) & =0.3\times log_{2}\frac{3}{0.3}+0.7\times log_{2}\frac{2}{0.7}\\ \; & =0.9966+1.0602=2.0568\\ C_{2}:H_{B\&F}\left(m_{2}\right) & =0.3\times log_{2}\frac{3^{4}}{0.3}+0.7\times log_{2}\frac{2^{4}}{0.7}\\ \; & =2.4230+3.1602=5.5832\\ C_{2}:H_{P}\left(m_{2}\right) & =0.3\times log_{2}\frac{3}{0.3}+0.7\times log_{2}\frac{2}{0.7}\\ \; & =0.9966+1.0602=2.0568 \end{array}$$

We can draw the following conclusions:

$$H_{B\&F}\left(m_{1}\right)>H_{B\&F}\left(m_{2}\right),H_{P}\left(m_{1}\right)=H_{P}\left(m_{2}\right)$$

By comparison, we can conclude that the result of B&F is more reasonable. Because of $C_{2}$ has fewer focal elements and they have the same element $x_{3}$ in two BPAs, therefore, the uncertainty of $C_{1}$ should be bigger than the uncertainty of $C_{2}$.

From an overall view, as long as the focal elements for every BPA are equal, the results of Pal entropy keep constant, even if the number of focal elements on FOD is different. This is unreasonable. However, for the new belief entropy, it reflects the impact of the number of FODs on information uncertainty. Obviously, the degree of information uncertainty is proportional to FOD. Thus, the new definition proposed in this paper is more reasonable for the above Section 6.1.4.

#### 6.1.5. Example 5

In this example, we suppose a FOD that has ten focal elements, $X=\{x_{1},x_{2}\cdots x_{10}\}$ and four mass functions, $m\left(\left\{3,4,5\right\}\right)=0.05,m\left(\left\{7\right\}\right)=0.05,m\left(\left\{B_{i}\right\}\right)=0.8,m\left(X\right)=0.1$, where $B_{i}$ is the subset of $2^{X}$ and *i* is equal to the cardinality of *B*. We chose ten subsets of $2^{X}$ to assignment *B* and used Dubois entropy, Deng entropy, Pan–Deng entropy, and the new belief entropy for comparison. In Section 4, we already listed these definitions of entropy. When $B_{i}$ changes, their values can be calculated by MATLAB. The calculation results of these definitions are shown in the following Table 1.

**Figure 2:**
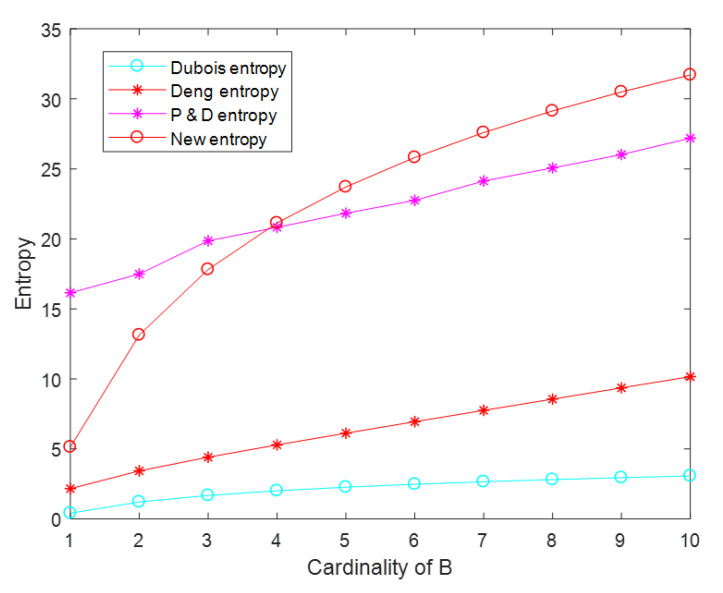
Comparison between the new belief entropy and other entropies.

Table 1 and Figure 2 show that the new belief entropy is larger than Deng entropy and Dubois entropy. On the other hand, the growth trend of the new belief entropy is slower than Deng entropy and Pan–Deng entropy and the same as Dubois entropy. For example, we chose $B_{1}$, $B_{2}$ and $B_{9}$, $B_{10}$ to illustrate the impact of each additional element in $B_{i}$ on uncertainty, under different cardinality of $B_{i}$. From the Table 1, we can get:

$$\begin{array}{cc} \; & H_{Dubois}\left(m_{2}\right)-H_{Dubois}\left(m_{1}\right)=0.8>H_{Dubois}\left(m_{10}\right)-H_{Dubois}\left(m_{9}\right)=0.1216 \end{array}$$

$$\begin{array}{cc} \; & H_{Deng}\left(m_{2}\right)-H_{Deng}\left(m_{1}\right)=1.2680\approx H_{Deng}\left(m_{10}\right)-H_{Deng}\left(m_{9}\right)=0.8012 \end{array}$$

$$\begin{array}{cc} \; & H_{P\&D}\left(m_{2}\right)-H_{P\&D}\left(m_{1}\right)=1.3473\approx H_{P\&D}\left(m_{10}\right)-H_{P\&D}\left(m_{9}\right)=1.1735 \end{array}$$

$$\begin{array}{cc} \; & H_{B\&F}\left(m_{2}\right)-H_{B\&F}\left(m_{1}\right)=8>H_{B\&F}\left(m_{10}\right)-H_{B\&F}\left(m_{9}\right)=1.2161 \end{array}$$

Where the P& D entropy in Figure 2 is the $H_{Bel}\left(m\right)$ we listed in Section 4.

Although the four entropy values in Figure 2 increased, their slopes were different. Deng entropy and Pan–Deng entropy increased linearly, while the slopes of Dubois entropy and the new entropy decreased with the increase of the cardinality of *B*. We believe that the growth trend of the latter was more reasonable. This was because the scale of B was an important indicator to measure the change of information uncertainty, which should change with the size of cardinality. With the same cardinality of $B_{i}$, our new belief entropy was larger than the Dubois entropy. It could well reflect the degree of uncertainty. Therefore, through comprehensive analysis, we considered that the new belief entropy was more accurate.

**Figure 3:**
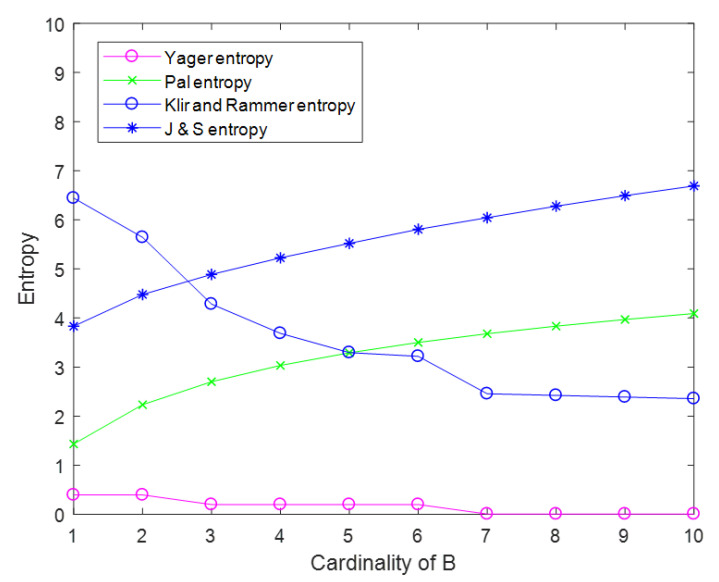
Results’ comparison of other entropies.

Yager entropy, Pal entropy, Klir and Rammer entropy, and Jiroušek and Shenoy entropy are plotted in Figure 3.

From Figure 3, it can be seen that these definitions kept a small value. The degrees of uncertainty measured by Klir and Rammer and Yager decreased visibly with the increasing of the elements in *B*. This was understandable. The uncertainty measures proposed by Pal and Jiroušek and Shenoy were nearly linear with the cardinality of *B*. They had the same growth trend as Deng entropy.

Where the J& S entropy in Figure 3 is the $H_{J\&S}\left(m\right)$ we listed in Section 4.

#### 6.1.6. Example 6

In recent years, much research has been modified based on Deng entropy theory [33,34,37]. In this example, we chose to compare W entropy and our new model.

Although W entropy takes into account the scale of FOD, the effect of the scale of FOD on W entropy is very limited [34]. As Equation (26) shows, the value of our new model would change exponentially with the scale of FOD. As they showed in their examples, when ϵ increased from zero to 10, the change trend of W entropy was almost the same as Deng entropy. However, as we demonstrated in Section 6.1.5, the growth trend of B&F entropy was different from Deng entropy. Therefore, we could see the effectiveness and superiority of the proposed entropy.

#### 6.1.7. Example Summary

Based on the examples proposed above, we list some typical cases that may affect the new belief entropy and compare it with other entropies. From Section 6.1.2 and Section 6.1.3, we could see that the new entropy was more sensitive to the vacuous BPA. Section 6.1.4 shows the limitations of the general entropy, and the new entropy could solve the problem caused by the different number of FODs. Section 6.1.5 reflected the change of the new entropy and other entropies as the number of elements increased. In Section 6.1.6, we made a simple comparison between W entropy and B&F entropy.

### 6.2. Simulation

Here, we use MATLAB to complete the test. This test could more intuitively feel how the new belief entropy changed with the different BPAs.

**Figure 4:**
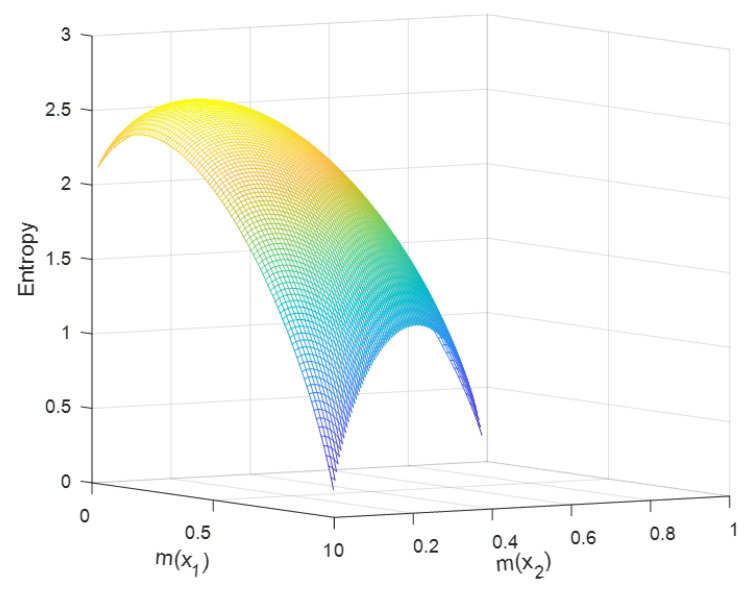
The value of the new belief entropy with changes of BPA.

**Figure 5:**
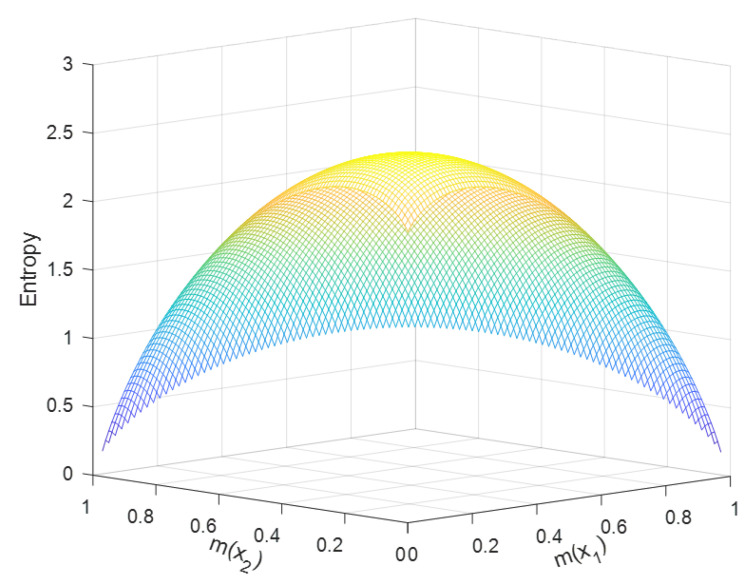
The value of the new belief entropy with changes of BPA.

We supposed an FOD $X=\{x_{1},x_{2}\}$. This FOD had three BPAs, $m\left(\left\{x_{1}\right\}\right)=p_{1}$, $m\left(\left\{x_{2}\right\}\right)=p_{2}$, and $m\left(\left\{x_{1},x_{2}\right\}\right)=1-p_{1}-p_{2}$. $p_{1}$ and $p_{2}$ can take any value from zero to one. However, according to D-S theory in Section 2, we limited the value of these BPAs, where $m\left(\left\{x_{1}\right\}\right)+m\left(\left\{x_{2}\right\}\right)=p_{1}+p_{2}\leq 1$. Obviously, $m(\left\{x_{1},x_{2}\right\})$ exists only when $m\left(\left\{x_{1}\right\}\right)+m\left(\left\{x_{2}\right\}\right)<1$. The simulation results are as Figure 4 and Figure 5 show, where the x-axis is $m(\left\{x_{1}\right\})$, the y-axis is $m(\left\{x_{2}\right\})$, and the z-axis means the value of the new entropy.

When $p_{1}+p_{2}=1$, the max value of the new entropy $H_{B\&F}\left(m\right)=1$, where $p_{1}=p_{2}=0.5$. When considering the BPA $m(\left\{x_{1},x_{2}\right\})$, $m(x_{1})=0.17$, $m(x_{2})=0.16$ and $m(\left\{x_{1},x_{2}\right\})=0.67$ could get the max value of the new entropy, $H_{B\&F(max)}\left(m\right)=2.585$. From this max value result, we obtained that the new definition did not satisfy the maximum entropy property.

Analysis: These simulation results suggested that the main trend of the new entropy was changing with different BPAs. It also indicated that the new entropy increased as the vacuous BPA increased, when $p_{1}+p_{2}<1$, which was reasonable. Therefore, the new entropy could reflect well the degree of measurement of information uncertainty.

## 7. Conclusions and Discussion

First of all, we reviewed some earlier definitions proposed by Hartley, Shannon, Yager, Nguyen, Lamata and Moral, Jiroušek and Shenoy, Klir and Ramer, Dubois, Nikhil R. Pal, Joussemle, Deng, and Pan–Deng. However, none of them reflected the number of FODs’ effect on uncertainty.

We discussed an open issue, which was how to measure information uncertainty. Our principle was to include as much known information as possible under D-S theory. Thus, in this paper, we considered the cardinality of FOD and defined a new model to measure uncertainty. Meanwhile, some properties of the new entropy were discussed. The result of the examples and simulation proved that the new entropy could be more effective and accurate when compared with other entropies.

When the target belonged to the set of clusters and the total number of targets could not be determined, our method could get the information uncertainty from the target accurately. Compared with traditional methods, the new entropy was easy to calculate. This meant that in the same time, it could process more data. In future work, we will apply it to solve practical problems and improve it in real applications.

## Figures and Tables

**Table 1:** The value of different definitions when $B_{i}$ changes.

Cases	Dubois Entropy	Deng Entropy	Pan–Deng Entropy	New Entropy
$B_{1}=\left\{1\right\}$	0.4114	2.6623	16.1443	5.1363
$B_{2}=\left\{1,2\right\}$	1.2114	3.9303	17.4916	13.1363
$B_{3}=\left\{1,2,3\right\}$	1.6794	4.9082	19.8608	17.8160
$B_{4}=\left\{1,2\ldots 4\right\}$	2.0114	5.7878	20.8229	21.1363
$B_{5}=\left\{1,2\ldots 5\right\}$	2.2690	6.6256	21.8314	23.7118
$B_{6}=\left\{1,2\ldots 6\right\}$	2.4794	7.4441	22.7521	25.8160
$B_{7}=\left\{1,2\ldots 7\right\}$	2.6573	8.2532	24.1331	27.5952
$B_{8}=\left\{1,2\ldots 8\right\}$	2.8114	9.0578	25.0685	29.1363
$B_{9}=\left\{1,2\ldots 9\right\}$	2.9474	9.8600	26.0212	30.4957
$B_{10}=\left\{1,2\ldots 10\right\}$	3.0690	10.6612	27.1947	31.7118
